# *cis*-(Pyridin-2-ylcarbonimidodithio­ato-κ^2^ *S*,*S*′)bis­(triphenyl­phosphane-κ*P*)palladium(II)

**DOI:** 10.1107/S1600536810050518

**Published:** 2010-12-08

**Authors:** Vladimir V. Bon, Svitlana I. Orysyk, Vasily I. Pekhnyo

**Affiliations:** a Institute of General and Inorganic Chemistry, NAS Ukraine, Kyiv, prosp. Palladina 32/34, 03680, Ukraine

## Abstract

The title compound, [Pd(C_6_H_4_N_2_S_2_)(C_18_H_15_P)_2_], was obtained as a minor product from the reaction of *trans*-PdCl_2_(PPh_3_)_2_ with piperazine-1,4-dicarbothioic acid bis­(pyridin-2-yl)amide. The Pd^II^ atom displays a slightly distorted square-planar PdP_2_S_2_ geometry with a bidentately coordinated pyridin-2-ylcarbonimidodithio­ate ligand and two triphenyl­phosphine mol­ecules, coordinated in *cis* positions. The crystal structure features weak π–π [centroid–centroid distance =3.7327(15) Å] and C–H⋯π inter­actions and contains an almost spherically shaped void of 50.4 Å^3^ per unit cell.

## Related literature

For the biological activity of Pd compounds, see: Garoufis *et al.* (2009 ▶). For related structures, see: Ahmed *et al.* (1977 ▶). For bond-length data, see: Allen *et al.* (1987 ▶).
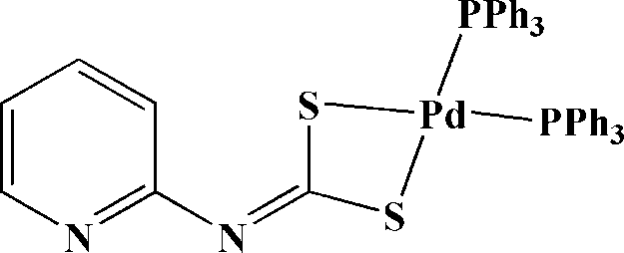


## Experimental

### 

#### Crystal data

[Pd(C_6_H_4_N_2_S_2_)(C_18_H_15_P)_2_]
*M* *_r_* = 799.17
Triclinic, 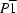

*a* = 10.9163 (10) Å
*b* = 12.7235 (12) Å
*c* = 15.7104 (14) Å
α = 102.609 (1)°
β = 107.672 (1)°
γ = 108.567 (1)°
*V* = 1847.2 (3) Å^3^
*Z* = 2
Mo *K*α radiation
μ = 0.74 mm^−1^
*T* = 173 K
0.50 × 0.30 × 0.16 mm

#### Data collection

Bruker APEXII CCD diffractometer
Absorption correction: multi-scan (*SADABS*; Bruker, 2005 ▶) *T*_min_ = 0.712, *T*_max_ = 0.890
30024 measured reflections
7568 independent reflections
6811 reflections with *I* > 2σ(*I*)
*R*_int_ = 0.025

#### Refinement

*R*[*F* ^2^ > 2σ(*F* ^2^)] = 0.023
*wR*(*F* ^2^) = 0.055
*S* = 1.06
7568 reflections
442 parameters
H-atom parameters constrained
Δρ_max_ = 0.43 e Å^−3^
Δρ_min_ = −0.54 e Å^−3^

### 

Data collection: *APEX2* (Bruker, 2005 ▶); cell refinement: *SAINT* (Bruker, 2005 ▶); data reduction: *SAINT*; program(s) used to solve structure: *SHELXS97* (Sheldrick, 2008 ▶); program(s) used to refine structure: *SHELXL97* (Sheldrick, 2008 ▶); molecular graphics: *PLATON* (Spek, 2009 ▶); software used to prepare material for publication: *publCIF* (Westrip, 2010 ▶).

## Supplementary Material

Crystal structure: contains datablocks I, global. DOI: 10.1107/S1600536810050518/im2253sup1.cif

Structure factors: contains datablocks I. DOI: 10.1107/S1600536810050518/im2253Isup2.hkl

Additional supplementary materials:  crystallographic information; 3D view; checkCIF report

## Figures and Tables

**Table 1:** Hydrogen-bond geometry (Å, °) *Cg*1, *Cg*2, *Cg*3, *Cg*4 and *Cg*5 are the centroids of the N2/C2/C6/C5/C4/C3, C7–C12, C13–C18, C19–C24 and C25–C30 rings, respectively.

*D*—H⋯*A*	*D*—H	H⋯*A*	*D*⋯*A*	*D*—H⋯*A*
C15—H15⋯*Cg*1^i^	0.95	2.73	3.4991 (3)	139
C20—H20⋯*Cg*2^ii^	0.95	2.93	3.7152 (2)	141
C9—H9⋯*Cg*3^ii^	0.95	2.70	3.4657 (2)	138
C4—H4⋯*Cg*4^iii^	0.95	2.72	3.5712 (2)	150
C35—H35⋯*Cg*5^iv^	0.95	2.98	3.7681 (2)	141

Symmetry codes: (i) 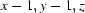; (ii) 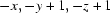; (iii) 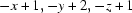; (iv) 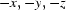.

**Fig. 1.**
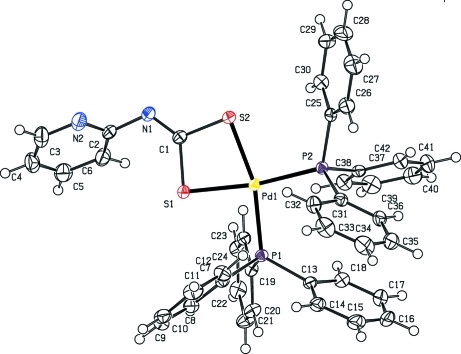
General view of title compound. Thermal ellipsoids are given at 50% probability.

**Fig. 2.**
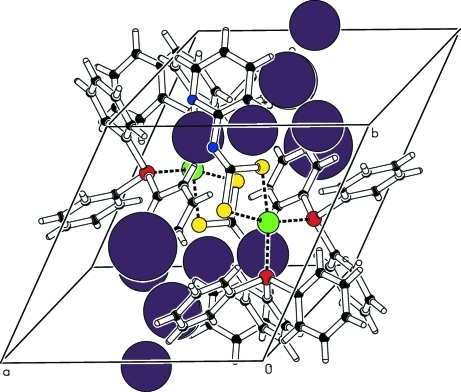
Packing diagram of title compound. Dark spheres indicate voids.

## supplementary crystallographic information

### Comment

Structural investigation of palladium coordination compounds with carbothioamide derivatives has an actuality due to their potential biological activity (Garoufis *et al.*, 2009). The current paper reports a structural study of a coordination compound obtained as a minor product as a result of the decomposition of the initially used organic ligand molecule in DMF solution.

The asymmetric unit of crystal structure contains one molecule of the complex, in which palladium displays a slightly distorted square-planar PdP_2_S_2_ coordination geometry (Fig. 1). Mean deviation from the plane Pd1/P1/P2/S1/S2 is 0.0455 Å (maximum deviation for S1 = -0.0646 (5) Å). The values of Pd–P and Pd–S bond lengths correspond to those in related structures (Ahmed *et al.*, 1977). S1–Pd1–S2 strongly deviates from 90° due to steric strain of the four-membered ring. The opposite angle P1–Pd1–P2 has a value of 99.77 (2)° that can be explained by repulsion of bulky phenyl rings of triphenylphosphine moieties. The organic ligand molecule coordinates to palladium bidentantly as a dianion. Two triphenylphosphine ligands coordinate to the palladium in a *cis-*conformation, although there is a *trans*-conformation in the initial reagent. The bonds C1–S1 and C2–S2 correspond to C–S single bonds (Allen *et al.*, 1987). At the same time, C1—N1 shows a value of 1.275 (2) Å corresponding to a classical C=N double bond. The ligand molecule contains two planar fragments C1/N1/S1/S2 and N1/N2/C2–C6, creating a dihedral angle of 58.22 (8)°. The crystal structure of the title compound shows a large number of weak π–π and C–H···π interactions and contains an almost spherically shaped void of 50.4 Å^3^ per unit cell (Fig. 2).

### Experimental

20 ml (10 ^-2^ mol/*L*) DMF solution of piperazine-1,4-dicarbothioic acid bis-pyridin-2-ylamide was stirred with 20 ml (2x10 ^-2^ mol/*L*) solution of PdCl_2_(PPh_3_)_2 _in chloroform. As a result of slow evaporation (1 month) several yellow prismatic crystals of the title compound were obtained from reaction mixture (yield ~5%).

### Refinement

All H atoms were placed in calculated positions with C–H = 0.95 Å and *U*_iso_(H) = 1.2*U*_iso_(C).

### Crystal data

**Table:**

[Pd(C_6_H_4_N_2_S_2_)(C_18_H_15_P)_2_]	*Z* = 2
*M**_r_* = 799.17	*F*(000) = 816
Triclinic, *P*1	*D*_x_ = 1.437 Mg m^−^^3^
Hall symbol: -P 1	Melting point: 558 K
*a* = 10.9163 (10) Å	Mo *K*α radiation, λ = 0.71073 Å
*b* = 12.7235 (12) Å	Cell parameters from 9826 reflections
*c* = 15.7104 (14) Å	θ = 2.7–26.5°
α = 102.609 (1)°	µ = 0.74 mm^−^^1^
β = 107.672 (1)°	*T* = 173 K
γ = 108.567 (1)°	Block, yellow
*V* = 1847.2 (3) Å^3^	0.50 × 0.30 × 0.16 mm

### Data collection

**Table:**

Bruker APEXII CCD diffractometer	7568 independent reflections
Radiation source: fine-focus sealed tube	6811 reflections with *I* > 2σ(*I*)
graphite	*R*_int_ = 0.025
φ and ω scans	θ_max_ = 26.5°, θ_min_ = 1.5°
Absorption correction: multi-scan (*SADABS*; Bruker, 2005)	*h* = −13→13
*T*_min_ = 0.712, *T*_max_ = 0.890	*k* = −15→15
30024 measured reflections	*l* = −19→19

### Refinement

**Table:**

Refinement on *F*^2^	Primary atom site location: structure-invariant direct methods
Least-squares matrix: full	Secondary atom site location: difference Fourier map
*R*[*F*^2^ > 2σ(*F*^2^)] = 0.023	Hydrogen site location: inferred from neighbouring sites
*wR*(*F*^2^) = 0.055	H-atom parameters constrained
*S* = 1.06	*w* = 1/[σ^2^(*F*_o_^2^) + (0.0192*P*)^2^ + 1.2908*P*] where *P* = (*F*_o_^2^ + 2*F*_c_^2^)/3
7568 reflections	(Δ/σ)_max_ = 0.001
442 parameters	Δρ_max_ = 0.43 e Å^−^^3^
0 restraints	Δρ_min_ = −0.54 e Å^−^^3^

### Special details

**Table:**

Geometry. All e.s.d.'s (except the e.s.d. in the dihedral angle between two l.s. planes) are estimated using the full covariance matrix. The cell e.s.d.'s are taken into account individually in the estimation of e.s.d.'s in distances, angles and torsion angles; correlations between e.s.d.'s in cell parameters are only used when they are defined by crystal symmetry. An approximate (isotropic) treatment of cell e.s.d.'s is used for estimating e.s.d.'s involving l.s. planes.
Refinement. Refinement of *F*^2^ against ALL reflections. The weighted *R*-factor *wR* and goodness of fit *S* are based on *F*^2^, conventional *R*-factors *R* are based on *F*, with *F* set to zero for negative *F*^2^. The threshold expression of *F*^2^ > σ(*F*^2^) is used only for calculating *R*-factors(gt) *etc*. and is not relevant to the choice of reflections for refinement. *R*-factors based on *F*^2^ are statistically about twice as large as those based on *F*, and *R*- factors based on ALL data will be even larger.

### Fractional atomic coordinates and isotropic or equivalent isotropic displacement parameters (Å^2^)

**Table:**

	*x*	*y*	*z*	*U*_iso_*/*U*_eq_	
Pd1	0.261679 (13)	0.490049 (11)	0.242076 (9)	0.01556 (4)	
S1	0.40275 (5)	0.68151 (4)	0.35196 (3)	0.02194 (10)	
S2	0.44221 (5)	0.56216 (4)	0.19302 (3)	0.02078 (10)	
P1	0.10786 (5)	0.47339 (4)	0.31797 (3)	0.01678 (9)	
P2	0.15254 (5)	0.30268 (4)	0.12882 (3)	0.01660 (9)	
N1	0.61298 (16)	0.78937 (13)	0.29662 (11)	0.0242 (3)	
N2	0.79863 (18)	0.93668 (15)	0.43303 (12)	0.0315 (4)	
C1	0.50723 (18)	0.69887 (16)	0.28491 (13)	0.0198 (4)	
C2	0.6625 (2)	0.89572 (16)	0.37398 (13)	0.0244 (4)	
C3	0.8521 (2)	1.03808 (18)	0.50638 (15)	0.0364 (5)	
H3	0.9481	1.0677	0.5491	0.044*	
C4	0.7757 (3)	1.10161 (18)	0.52328 (15)	0.0382 (5)	
H4	0.8177	1.1726	0.5767	0.046*	
C5	0.6371 (3)	1.06015 (18)	0.46105 (16)	0.0380 (5)	
H5	0.5820	1.1026	0.4707	0.046*	
C6	0.5785 (2)	0.95570 (17)	0.38411 (15)	0.0324 (5)	
H6	0.4835	0.9260	0.3396	0.039*	
C7	0.21273 (18)	0.52223 (15)	0.44574 (12)	0.0187 (4)	
C8	0.2197 (2)	0.61859 (16)	0.51214 (13)	0.0239 (4)	
H8	0.1648	0.6604	0.4921	0.029*	
C9	0.3069 (2)	0.65392 (18)	0.60798 (14)	0.0297 (4)	
H9	0.3128	0.7209	0.6529	0.036*	
C10	0.3851 (2)	0.59247 (19)	0.63835 (14)	0.0306 (5)	
H10	0.4415	0.6152	0.7042	0.037*	
C11	0.3810 (2)	0.4978 (2)	0.57281 (15)	0.0321 (5)	
H11	0.4356	0.4560	0.5935	0.039*	
C12	0.2972 (2)	0.46390 (18)	0.47686 (14)	0.0268 (4)	
H12	0.2971	0.4004	0.4318	0.032*	
C13	−0.03915 (19)	0.33285 (15)	0.28912 (13)	0.0208 (4)	
C14	−0.0328 (2)	0.25909 (16)	0.34274 (14)	0.0262 (4)	
H14	0.0485	0.2836	0.3996	0.031*	
C15	−0.1447 (2)	0.14992 (18)	0.31339 (16)	0.0367 (5)	
H15	−0.1393	0.1002	0.3503	0.044*	
C16	−0.2634 (2)	0.11321 (19)	0.23120 (17)	0.0405 (6)	
H16	−0.3392	0.0383	0.2113	0.049*	
C17	−0.2717 (2)	0.18551 (19)	0.17795 (16)	0.0376 (5)	
H17	−0.3537	0.1603	0.1214	0.045*	
C18	−0.1609 (2)	0.29515 (17)	0.20653 (14)	0.0277 (4)	
H18	−0.1680	0.3448	0.1697	0.033*	
C19	0.01908 (18)	0.57093 (15)	0.29947 (12)	0.0187 (4)	
C20	−0.07535 (19)	0.57915 (17)	0.34150 (14)	0.0250 (4)	
H20	−0.0926	0.5343	0.3809	0.030*	
C21	−0.1439 (2)	0.65267 (18)	0.32576 (15)	0.0285 (4)	
H21	−0.2062	0.6592	0.3556	0.034*	
C22	−0.1218 (2)	0.71635 (17)	0.26679 (14)	0.0285 (4)	
H22	−0.1693	0.7661	0.2559	0.034*	
C23	−0.0304 (2)	0.70755 (17)	0.22360 (13)	0.0259 (4)	
H23	−0.0164	0.7503	0.1823	0.031*	
C24	0.04113 (19)	0.63604 (15)	0.24065 (12)	0.0206 (4)	
H24	0.1053	0.6316	0.2120	0.025*	
C25	0.25221 (18)	0.26491 (16)	0.06117 (12)	0.0214 (4)	
C26	0.2939 (2)	0.17213 (18)	0.06223 (14)	0.0295 (4)	
H26	0.2721	0.1261	0.1001	0.035*	
C27	0.3676 (2)	0.1469 (2)	0.00776 (17)	0.0425 (6)	
H27	0.3943	0.0828	0.0077	0.051*	
C28	0.4017 (2)	0.2146 (2)	−0.04599 (17)	0.0470 (6)	
H28	0.4528	0.1975	−0.0824	0.056*	
C29	0.3620 (2)	0.3075 (2)	−0.04720 (15)	0.0408 (6)	
H29	0.3865	0.3544	−0.0839	0.049*	
C30	0.2863 (2)	0.33189 (18)	0.00545 (14)	0.0298 (4)	
H30	0.2575	0.3946	0.0035	0.036*	
C31	0.12398 (19)	0.19049 (15)	0.18454 (12)	0.0195 (4)	
C32	0.2376 (2)	0.21068 (17)	0.26675 (14)	0.0273 (4)	
H32	0.3220	0.2806	0.2909	0.033*	
C33	0.2284 (2)	0.12971 (19)	0.31339 (15)	0.0358 (5)	
H33	0.3061	0.1440	0.3693	0.043*	
C34	0.1051 (3)	0.02747 (18)	0.27813 (15)	0.0372 (5)	
H34	0.0989	−0.0288	0.3094	0.045*	
C35	−0.0081 (2)	0.00757 (17)	0.19791 (15)	0.0317 (5)	
H35	−0.0925	−0.0622	0.1745	0.038*	
C36	0.0001 (2)	0.08889 (16)	0.15087 (13)	0.0238 (4)	
H36	−0.0788	0.0750	0.0959	0.029*	
C37	−0.01405 (18)	0.27074 (15)	0.03346 (12)	0.0189 (4)	
C38	−0.0635 (2)	0.35919 (17)	0.03277 (13)	0.0255 (4)	
H38	−0.0109	0.4344	0.0822	0.031*	
C39	−0.1891 (2)	0.33812 (19)	−0.03965 (15)	0.0330 (5)	
H39	−0.2220	0.3988	−0.0395	0.040*	
C40	−0.2660 (2)	0.2292 (2)	−0.11178 (14)	0.0330 (5)	
H40	−0.3526	0.2146	−0.1606	0.040*	
C41	−0.2168 (2)	0.14114 (19)	−0.11288 (14)	0.0321 (5)	
H41	−0.2694	0.0664	−0.1628	0.038*	
C42	−0.0908 (2)	0.16209 (17)	−0.04121 (13)	0.0251 (4)	
H42	−0.0566	0.1021	−0.0430	0.030*	

### Atomic displacement parameters (Å^2^)

**Table:**

	*U*^11^	*U*^22^	*U*^33^	*U*^12^	*U*^13^	*U*^23^
Pd1	0.01550 (7)	0.01378 (7)	0.01700 (7)	0.00506 (5)	0.00705 (5)	0.00543 (5)
S1	0.0217 (2)	0.0161 (2)	0.0238 (2)	0.00335 (18)	0.01150 (18)	0.00261 (18)
S2	0.0189 (2)	0.0201 (2)	0.0219 (2)	0.00547 (17)	0.01043 (17)	0.00524 (17)
P1	0.0178 (2)	0.0159 (2)	0.0188 (2)	0.00725 (17)	0.00877 (17)	0.00762 (17)
P2	0.0173 (2)	0.0147 (2)	0.0171 (2)	0.00594 (17)	0.00677 (17)	0.00551 (17)
N1	0.0203 (8)	0.0208 (8)	0.0279 (8)	0.0048 (6)	0.0110 (7)	0.0060 (7)
N2	0.0281 (9)	0.0266 (9)	0.0310 (9)	0.0047 (7)	0.0078 (7)	0.0094 (7)
C1	0.0173 (8)	0.0210 (9)	0.0232 (9)	0.0092 (7)	0.0086 (7)	0.0090 (7)
C2	0.0253 (10)	0.0192 (9)	0.0263 (10)	0.0030 (8)	0.0129 (8)	0.0095 (8)
C3	0.0359 (12)	0.0278 (11)	0.0281 (11)	0.0030 (9)	0.0030 (9)	0.0074 (9)
C4	0.0502 (14)	0.0218 (10)	0.0295 (11)	0.0045 (10)	0.0127 (10)	0.0056 (9)
C5	0.0462 (13)	0.0241 (11)	0.0479 (13)	0.0136 (10)	0.0255 (11)	0.0123 (10)
C6	0.0270 (10)	0.0242 (10)	0.0390 (12)	0.0056 (8)	0.0120 (9)	0.0077 (9)
C7	0.0180 (8)	0.0205 (9)	0.0191 (9)	0.0068 (7)	0.0090 (7)	0.0092 (7)
C8	0.0249 (9)	0.0249 (10)	0.0248 (9)	0.0109 (8)	0.0125 (8)	0.0096 (8)
C9	0.0295 (10)	0.0316 (11)	0.0241 (10)	0.0088 (9)	0.0135 (8)	0.0049 (8)
C10	0.0200 (9)	0.0452 (12)	0.0217 (10)	0.0072 (9)	0.0076 (8)	0.0133 (9)
C11	0.0247 (10)	0.0450 (13)	0.0342 (11)	0.0189 (9)	0.0116 (9)	0.0212 (10)
C12	0.0283 (10)	0.0307 (10)	0.0264 (10)	0.0169 (9)	0.0119 (8)	0.0108 (8)
C13	0.0228 (9)	0.0169 (9)	0.0248 (9)	0.0067 (7)	0.0144 (7)	0.0065 (7)
C14	0.0328 (10)	0.0215 (9)	0.0297 (10)	0.0110 (8)	0.0182 (9)	0.0110 (8)
C15	0.0529 (14)	0.0217 (10)	0.0458 (13)	0.0119 (10)	0.0347 (12)	0.0144 (9)
C16	0.0418 (13)	0.0217 (10)	0.0479 (14)	−0.0025 (9)	0.0299 (11)	0.0007 (10)
C17	0.0276 (11)	0.0348 (12)	0.0337 (11)	0.0005 (9)	0.0132 (9)	−0.0012 (9)
C18	0.0254 (10)	0.0272 (10)	0.0275 (10)	0.0069 (8)	0.0125 (8)	0.0078 (8)
C19	0.0165 (8)	0.0170 (8)	0.0225 (9)	0.0076 (7)	0.0066 (7)	0.0073 (7)
C20	0.0231 (9)	0.0268 (10)	0.0313 (10)	0.0111 (8)	0.0143 (8)	0.0154 (8)
C21	0.0247 (10)	0.0317 (11)	0.0374 (11)	0.0154 (9)	0.0172 (9)	0.0147 (9)
C22	0.0261 (10)	0.0264 (10)	0.0357 (11)	0.0153 (8)	0.0100 (8)	0.0125 (9)
C23	0.0311 (10)	0.0239 (10)	0.0265 (10)	0.0130 (8)	0.0114 (8)	0.0137 (8)
C24	0.0213 (9)	0.0196 (9)	0.0213 (9)	0.0082 (7)	0.0095 (7)	0.0067 (7)
C25	0.0164 (8)	0.0219 (9)	0.0187 (9)	0.0040 (7)	0.0060 (7)	0.0012 (7)
C26	0.0232 (10)	0.0320 (11)	0.0302 (10)	0.0130 (8)	0.0096 (8)	0.0046 (9)
C27	0.0293 (11)	0.0478 (14)	0.0447 (13)	0.0216 (11)	0.0131 (10)	−0.0004 (11)
C28	0.0290 (12)	0.0623 (16)	0.0395 (13)	0.0137 (11)	0.0205 (10)	−0.0025 (12)
C29	0.0323 (12)	0.0491 (14)	0.0282 (11)	0.0021 (10)	0.0178 (9)	0.0043 (10)
C30	0.0291 (10)	0.0294 (11)	0.0247 (10)	0.0066 (9)	0.0121 (8)	0.0047 (8)
C31	0.0246 (9)	0.0166 (8)	0.0206 (9)	0.0106 (7)	0.0112 (7)	0.0066 (7)
C32	0.0285 (10)	0.0232 (10)	0.0269 (10)	0.0100 (8)	0.0082 (8)	0.0076 (8)
C33	0.0459 (13)	0.0358 (12)	0.0291 (11)	0.0225 (10)	0.0108 (10)	0.0153 (9)
C34	0.0621 (15)	0.0260 (11)	0.0350 (12)	0.0225 (11)	0.0241 (11)	0.0188 (9)
C35	0.0424 (12)	0.0187 (9)	0.0343 (11)	0.0084 (9)	0.0202 (10)	0.0096 (8)
C36	0.0275 (10)	0.0175 (9)	0.0243 (9)	0.0075 (8)	0.0108 (8)	0.0056 (7)
C37	0.0192 (8)	0.0213 (9)	0.0187 (8)	0.0081 (7)	0.0100 (7)	0.0089 (7)
C38	0.0298 (10)	0.0246 (10)	0.0237 (9)	0.0131 (8)	0.0102 (8)	0.0094 (8)
C39	0.0363 (11)	0.0378 (12)	0.0333 (11)	0.0237 (10)	0.0128 (9)	0.0168 (9)
C40	0.0266 (10)	0.0468 (13)	0.0247 (10)	0.0157 (10)	0.0063 (8)	0.0157 (9)
C41	0.0298 (11)	0.0331 (11)	0.0208 (10)	0.0079 (9)	0.0039 (8)	0.0033 (8)
C42	0.0272 (10)	0.0237 (10)	0.0225 (9)	0.0110 (8)	0.0086 (8)	0.0057 (8)

### Geometric parameters (Å, °)

**Table:**

Pd1—P2	2.3067 (5)	C18—H18	0.9500
Pd1—P1	2.3206 (5)	C19—C24	1.393 (2)
Pd1—S2	2.3236 (5)	C19—C20	1.400 (3)
Pd1—S1	2.3339 (5)	C20—C21	1.389 (3)
S1—C1	1.7691 (19)	C20—H20	0.9500
S2—C1	1.7632 (18)	C21—C22	1.383 (3)
P1—C19	1.8212 (18)	C21—H21	0.9500
P1—C7	1.8257 (17)	C22—C23	1.385 (3)
P1—C13	1.8318 (18)	C22—H22	0.9500
P2—C37	1.8237 (18)	C23—C24	1.393 (3)
P2—C31	1.8246 (18)	C23—H23	0.9500
P2—C25	1.8320 (19)	C24—H24	0.9500
N1—C1	1.275 (2)	C25—C26	1.395 (3)
N1—C2	1.421 (2)	C25—C30	1.397 (3)
N2—C3	1.340 (3)	C26—C27	1.394 (3)
N2—C2	1.342 (2)	C26—H26	0.9500
C2—C6	1.389 (3)	C27—C28	1.378 (4)
C3—C4	1.375 (3)	C27—H27	0.9500
C3—H3	0.9500	C28—C29	1.384 (4)
C4—C5	1.375 (3)	C28—H28	0.9500
C4—H4	0.9500	C29—C30	1.389 (3)
C5—C6	1.387 (3)	C29—H29	0.9500
C5—H5	0.9500	C30—H30	0.9500
C6—H6	0.9500	C31—C36	1.391 (2)
C7—C8	1.390 (3)	C31—C32	1.397 (3)
C7—C12	1.400 (3)	C32—C33	1.385 (3)
C8—C9	1.392 (3)	C32—H32	0.9500
C8—H8	0.9500	C33—C34	1.388 (3)
C9—C10	1.381 (3)	C33—H33	0.9500
C9—H9	0.9500	C34—C35	1.377 (3)
C10—C11	1.382 (3)	C34—H34	0.9500
C10—H10	0.9500	C35—C36	1.393 (3)
C11—C12	1.387 (3)	C35—H35	0.9500
C11—H11	0.9500	C36—H36	0.9500
C12—H12	0.9500	C37—C38	1.395 (3)
C13—C14	1.395 (3)	C37—C42	1.395 (2)
C13—C18	1.399 (3)	C38—C39	1.390 (3)
C14—C15	1.390 (3)	C38—H38	0.9500
C14—H14	0.9500	C39—C40	1.380 (3)
C15—C16	1.378 (3)	C39—H39	0.9500
C15—H15	0.9500	C40—C41	1.388 (3)
C16—C17	1.377 (3)	C40—H40	0.9500
C16—H16	0.9500	C41—C42	1.388 (3)
C17—C18	1.390 (3)	C41—H41	0.9500
C17—H17	0.9500	C42—H42	0.9500
			
P2—Pd1—P1	99.768 (17)	C17—C18—H18	119.8
P2—Pd1—S2	96.202 (17)	C13—C18—H18	119.8
P1—Pd1—S2	163.859 (17)	C24—C19—C20	118.93 (16)
P2—Pd1—S1	171.187 (17)	C24—C19—P1	120.20 (14)
P1—Pd1—S1	88.614 (17)	C20—C19—P1	120.84 (14)
S2—Pd1—S1	75.557 (17)	C21—C20—C19	120.29 (18)
C1—S1—Pd1	87.98 (6)	C21—C20—H20	119.9
C1—S2—Pd1	88.45 (6)	C19—C20—H20	119.9
C19—P1—C7	107.41 (8)	C22—C21—C20	120.26 (19)
C19—P1—C13	102.38 (8)	C22—C21—H21	119.9
C7—P1—C13	105.76 (8)	C20—C21—H21	119.9
C19—P1—Pd1	110.53 (6)	C21—C22—C23	120.00 (18)
C7—P1—Pd1	107.71 (6)	C21—C22—H22	120.0
C13—P1—Pd1	122.17 (6)	C23—C22—H22	120.0
C37—P2—C31	108.92 (8)	C22—C23—C24	120.09 (18)
C37—P2—C25	101.60 (8)	C22—C23—H23	120.0
C31—P2—C25	103.24 (8)	C24—C23—H23	120.0
C37—P2—Pd1	115.41 (6)	C23—C24—C19	120.40 (17)
C31—P2—Pd1	110.71 (6)	C23—C24—H24	119.8
C25—P2—Pd1	115.92 (6)	C19—C24—H24	119.8
C1—N1—C2	119.58 (16)	C26—C25—C30	118.96 (18)
C3—N2—C2	117.30 (19)	C26—C25—P2	123.02 (15)
N1—C1—S2	122.57 (14)	C30—C25—P2	118.02 (15)
N1—C1—S1	129.67 (14)	C27—C26—C25	120.1 (2)
S2—C1—S1	107.75 (9)	C27—C26—H26	120.0
N2—C2—C6	122.92 (18)	C25—C26—H26	120.0
N2—C2—N1	114.58 (17)	C28—C27—C26	120.2 (2)
C6—C2—N1	122.40 (17)	C28—C27—H27	119.9
N2—C3—C4	123.7 (2)	C26—C27—H27	119.9
N2—C3—H3	118.2	C27—C28—C29	120.4 (2)
C4—C3—H3	118.2	C27—C28—H28	119.8
C3—C4—C5	118.5 (2)	C29—C28—H28	119.8
C3—C4—H4	120.7	C28—C29—C30	119.8 (2)
C5—C4—H4	120.7	C28—C29—H29	120.1
C4—C5—C6	119.4 (2)	C30—C29—H29	120.1
C4—C5—H5	120.3	C29—C30—C25	120.6 (2)
C6—C5—H5	120.3	C29—C30—H30	119.7
C5—C6—C2	118.2 (2)	C25—C30—H30	119.7
C5—C6—H6	120.9	C36—C31—C32	119.22 (17)
C2—C6—H6	120.9	C36—C31—P2	124.91 (14)
C8—C7—C12	118.79 (17)	C32—C31—P2	115.87 (14)
C8—C7—P1	123.31 (14)	C33—C32—C31	120.60 (19)
C12—C7—P1	117.77 (14)	C33—C32—H32	119.7
C7—C8—C9	120.08 (18)	C31—C32—H32	119.7
C7—C8—H8	120.0	C32—C33—C34	119.70 (19)
C9—C8—H8	120.0	C32—C33—H33	120.2
C10—C9—C8	120.55 (19)	C34—C33—H33	120.2
C10—C9—H9	119.7	C35—C34—C33	120.12 (19)
C8—C9—H9	119.7	C35—C34—H34	119.9
C9—C10—C11	119.91 (18)	C33—C34—H34	119.9
C9—C10—H10	120.0	C34—C35—C36	120.54 (19)
C11—C10—H10	120.0	C34—C35—H35	119.7
C10—C11—C12	119.90 (19)	C36—C35—H35	119.7
C10—C11—H11	120.1	C31—C36—C35	119.81 (18)
C12—C11—H11	120.1	C31—C36—H36	120.1
C11—C12—C7	120.68 (18)	C35—C36—H36	120.1
C11—C12—H12	119.7	C38—C37—C42	118.81 (17)
C7—C12—H12	119.7	C38—C37—P2	119.11 (14)
C14—C13—C18	118.55 (17)	C42—C37—P2	122.02 (14)
C14—C13—P1	123.23 (14)	C39—C38—C37	120.48 (18)
C18—C13—P1	118.14 (14)	C39—C38—H38	119.8
C15—C14—C13	120.31 (19)	C37—C38—H38	119.8
C15—C14—H14	119.8	C40—C39—C38	120.16 (19)
C13—C14—H14	119.8	C40—C39—H39	119.9
C16—C15—C14	120.5 (2)	C38—C39—H39	119.9
C16—C15—H15	119.7	C39—C40—C41	119.96 (18)
C14—C15—H15	119.7	C39—C40—H40	120.0
C17—C16—C15	119.82 (19)	C41—C40—H40	120.0
C17—C16—H16	120.1	C40—C41—C42	120.11 (19)
C15—C16—H16	120.1	C40—C41—H41	119.9
C16—C17—C18	120.3 (2)	C42—C41—H41	119.9
C16—C17—H17	119.8	C41—C42—C37	120.44 (18)
C18—C17—H17	119.8	C41—C42—H42	119.8
C17—C18—C13	120.42 (19)	C37—C42—H42	119.8
			
P1—Pd1—S1—C1	−173.50 (6)	C15—C16—C17—C18	0.2 (3)
S2—Pd1—S1—C1	3.32 (6)	C16—C17—C18—C13	0.6 (3)
P2—Pd1—S2—C1	179.85 (6)	C14—C13—C18—C17	−1.2 (3)
P1—Pd1—S2—C1	8.20 (9)	P1—C13—C18—C17	175.65 (16)
S1—Pd1—S2—C1	−3.33 (6)	C7—P1—C19—C24	121.46 (15)
P2—Pd1—P1—C19	−114.37 (6)	C13—P1—C19—C24	−127.42 (15)
S2—Pd1—P1—C19	57.20 (9)	Pd1—P1—C19—C24	4.21 (16)
S1—Pd1—P1—C19	68.37 (6)	C7—P1—C19—C20	−60.49 (16)
P2—Pd1—P1—C7	128.56 (6)	C13—P1—C19—C20	50.63 (16)
S2—Pd1—P1—C7	−59.86 (9)	Pd1—P1—C19—C20	−177.74 (13)
S1—Pd1—P1—C7	−48.70 (6)	C24—C19—C20—C21	−1.0 (3)
P2—Pd1—P1—C13	6.03 (7)	P1—C19—C20—C21	−179.10 (15)
S2—Pd1—P1—C13	177.61 (8)	C19—C20—C21—C22	1.4 (3)
S1—Pd1—P1—C13	−171.23 (7)	C20—C21—C22—C23	−0.4 (3)
P1—Pd1—P2—C37	70.58 (6)	C21—C22—C23—C24	−1.0 (3)
S2—Pd1—P2—C37	−107.07 (6)	C22—C23—C24—C19	1.4 (3)
P1—Pd1—P2—C31	−53.70 (6)	C20—C19—C24—C23	−0.4 (3)
S2—Pd1—P2—C31	128.65 (6)	P1—C19—C24—C23	177.72 (14)
P1—Pd1—P2—C25	−170.83 (7)	C37—P2—C25—C26	−115.11 (16)
S2—Pd1—P2—C25	11.52 (7)	C31—P2—C25—C26	−2.26 (17)
C2—N1—C1—S2	−178.05 (14)	Pd1—P2—C25—C26	118.96 (14)
C2—N1—C1—S1	0.8 (3)	C37—P2—C25—C30	64.24 (15)
Pd1—S2—C1—N1	−176.49 (15)	C31—P2—C25—C30	177.10 (14)
Pd1—S2—C1—S1	4.46 (8)	Pd1—P2—C25—C30	−61.69 (15)
Pd1—S1—C1—N1	176.59 (17)	C30—C25—C26—C27	−0.4 (3)
Pd1—S1—C1—S2	−4.44 (8)	P2—C25—C26—C27	178.91 (15)
C3—N2—C2—C6	2.3 (3)	C25—C26—C27—C28	1.2 (3)
C3—N2—C2—N1	178.74 (17)	C26—C27—C28—C29	−0.7 (3)
C1—N1—C2—N2	123.01 (19)	C27—C28—C29—C30	−0.5 (3)
C1—N1—C2—C6	−60.6 (3)	C28—C29—C30—C25	1.3 (3)
C2—N2—C3—C4	−0.6 (3)	C26—C25—C30—C29	−0.8 (3)
N2—C3—C4—C5	−0.9 (3)	P2—C25—C30—C29	179.85 (15)
C3—C4—C5—C6	0.6 (3)	C37—P2—C31—C36	8.05 (19)
C4—C5—C6—C2	1.1 (3)	C25—P2—C31—C36	−99.36 (17)
N2—C2—C6—C5	−2.6 (3)	Pd1—P2—C31—C36	135.96 (15)
N1—C2—C6—C5	−178.74 (18)	C37—P2—C31—C32	−172.34 (14)
C19—P1—C7—C8	−0.90 (18)	C25—P2—C31—C32	80.26 (15)
C13—P1—C7—C8	−109.69 (16)	Pd1—P2—C31—C32	−44.43 (16)
Pd1—P1—C7—C8	118.17 (15)	C36—C31—C32—C33	1.2 (3)
C19—P1—C7—C12	−176.58 (14)	P2—C31—C32—C33	−178.47 (16)
C13—P1—C7—C12	74.64 (16)	C31—C32—C33—C34	0.0 (3)
Pd1—P1—C7—C12	−57.50 (15)	C32—C33—C34—C35	−0.9 (3)
C12—C7—C8—C9	−1.5 (3)	C33—C34—C35—C36	0.6 (3)
P1—C7—C8—C9	−177.10 (14)	C32—C31—C36—C35	−1.5 (3)
C7—C8—C9—C10	−1.3 (3)	P2—C31—C36—C35	178.10 (15)
C8—C9—C10—C11	2.5 (3)	C34—C35—C36—C31	0.6 (3)
C9—C10—C11—C12	−0.8 (3)	C31—P2—C37—C38	126.65 (15)
C10—C11—C12—C7	−2.1 (3)	C25—P2—C37—C38	−124.84 (15)
C8—C7—C12—C11	3.2 (3)	Pd1—P2—C37—C38	1.44 (17)
P1—C7—C12—C11	179.03 (15)	C31—P2—C37—C42	−56.15 (17)
C19—P1—C13—C14	−137.46 (16)	C25—P2—C37—C42	52.37 (17)
C7—P1—C13—C14	−25.11 (18)	Pd1—P2—C37—C42	178.64 (13)
Pd1—P1—C13—C14	98.32 (16)	C42—C37—C38—C39	1.8 (3)
C19—P1—C13—C18	45.87 (16)	P2—C37—C38—C39	179.09 (16)
C7—P1—C13—C18	158.23 (15)	C37—C38—C39—C40	−0.1 (3)
Pd1—P1—C13—C18	−78.34 (16)	C38—C39—C40—C41	−1.0 (3)
C18—C13—C14—C15	0.9 (3)	C39—C40—C41—C42	0.4 (3)
P1—C13—C14—C15	−175.73 (15)	C40—C41—C42—C37	1.3 (3)
C13—C14—C15—C16	−0.1 (3)	C38—C37—C42—C41	−2.4 (3)
C14—C15—C16—C17	−0.4 (3)	P2—C37—C42—C41	−179.61 (15)

### Hydrogen-bond geometry (Å, °)

**Table:**

Cg1, Cg2, Cg3, Cg4 and Cg5 are the centroids of the N2/C2/C6/C5/C4/C3, C7–C12, C13–C18, C19–C24 and C25–C30 rings, respectively.

**Table:**

*D*—H···*A*	*D*—H	H···*A*	*D*···*A*	*D*—H···*A*
C15—H15···Cg1^i^	0.95	2.73	3.4991 (3)	139
C20—H20···Cg2^ii^	0.95	2.93	3.7152 (2)	141
C9—H9···Cg3^ii^	0.95	2.70	3.4657 (2)	138
C4—H4···Cg4^iii^	0.95	2.72	3.5712 (2)	150
C35—H35···Cg5^iv^	0.95	2.98	3.7681 (2)	141

Symmetry codes: (i) *x*−1, *y*−1, *z*; (ii) −*x*, −*y*+1, −*z*+1; (iii) −*x*+1, −*y*+2, −*z*+1; (iv) −*x*, −*y*, −*z*.
